# Lymphatic Drainage of Legs Reduces Edema of the Arms in Children with Lymphedema

**DOI:** 10.1155/2018/6038907

**Published:** 2018-02-18

**Authors:** Lívia Maria Pereira de Godoy, Paula Pereira de Godoy Capeletto, Maria de Fátima Guerreiro Godoy, Jose Maria Pereira de Godoy

**Affiliations:** ^1^General Clinics in the Medical School of Marilia and Research Group of Clínica Godoy, São Jose do Rio Preto, SP, Brazil; ^2^Medical School of Votuporanga and Research Group of Clínica Godoy, São Jose do Rio Preto, SP, Brazil; ^3^Medicine School in São José do Rio Preto (FAMERP) and Research Group in the Clínica Godoy, Sao Jose do Rio Preto, SP, Brazil; ^4^Cardiology and Cardiovascular Surgery Department of the Medical School in São Jose do Rio Preto (FAMERP) and CNPq (National Council for Research and Development), Sao Jose do Rio Preto, SP, Brazil

## Abstract

**Objective:**

The aim of the present study is to report on the reduction of edema of lymphedematous arms just by treating the lower limbs.

**Methods:**

A 16-year-old girl reported that she has started having right lower limb edema at the age of three. At age 13, she performed a lymphoscintigraphy that confirmed the diagnosis of primary lymphedema of the four limbs. Recently she sought treatment at the Clínica Godoy in São Jose do Rio Preto where she was submitted to intensive treatment for eight hours per day for five days using manual (Godoy & Godoy technique) and mechanical lymphatic therapy (RA Godoy^®^) of the lower limbs, cervical lymphatic therapy (cervical stimulation), and the continuous use of a grosgrain stocking.

**Results:**

At the end of treatment, reductions in the sizes of both arms and legs were noted even without the use of any specific therapy for the arms. After four years, the size of the arms was normal.

**Conclusion:**

Treatment of lymphedema of the legs has systemic repercussions that may lead to the reduction in swelling of other untreated regions of the body.

## 1. Introduction

Lymphedema is a clinical sign of impairment of the lymphatic system in respect to the formation and drainage of lymph. This leads to an accumulation of macromolecules and liquids in the interstitial space, which can affect any part of the body, but it is more common in the extremities [1].

Congenital lymphedema is a rare disease with a prevalence of 1.15/100,000 people [2]. One study showed that of 142 children with lymphedema, 58.7% were female and 41.3% were male. In this study, the onset in males occurred predominantly in childhood, whereas for the girls, the onset generally occurred in adolescence [3].

The diagnosis of lymphedema is clinical and by laboratory analysis. Early diagnosis is important due to the progressive character of lymphedema that can reach proportions that are difficult to revert and can limit the life of the patient [2]. The quality of life of the patient should always be considered during treatment. The main forms of therapies employed in the treatment of lymphedema are lymph drainage, restraint mechanisms, myolymphokinetic exercises and activities, and hygienic care. These therapies can be performed in isolation or association. In recent years, Godoy & Godoy have used an intensive form of lymphedema treatment, allowing reductions of around 50% of the limb volume with one week of treatment [4].

The therapeutic evolution of lymphedema treatment in adolescents has not frequently been discussed in the literature. The aim of the present study is to report on the reduction of edema of lymphedematous upper limbs just by treating the lower limbs.

## 2. Case Report

A 16-year-old female teenager reported that she began to present with edema of her right leg at the age of three at which time she sought treatment; however, the cause of the swelling was not diagnosed. At ten years of age, she also observed that her left leg was swollen, and at the age of 13, a lymphoscintigraphy was performed which detected lymphedema of all four limbs, arms, and legs. At 16 years of age, after erysipelas, the edema of the lower limbs worsened and clinically identifiable lymphedema of the right arm was observed, especially the hands, so she sought the Clínica Godoy for treatment. A volumetric evaluation was performed of all four limbs, and an intensive 8-hour treatment program was proposed. The adolescent was submitted to manual (Godoy & Godoy technique) [5] and mechanical lymphatic therapy (RA Godoy) [6] of the lower limbs, cervical lymphatic therapy (cervical stimulation) [7], and the continuous use of a grosgrain stocking [4].

A reduction in the size of all four limbs was observed at the end of the 5-day treatment program without using any therapies specifically to treat the arms (Table 1). Maintenance of the results also used the grosgrain stocking and mechanical lymphatic therapy (RA Godoy) of the legs at the patient's home.

In the maintenance phase, the patient was advised about the need of skin care. Moreover, she performed myolymphokinetic exercises of both the legs and arms, prioritizing activities that require little effort and did not involve impact or repetitive movements that could aggravate the edema. The patient was asked to return for monitoring by the care team every three months; however, she did not return. Only after four years, she returned to the service to treat an outbreak of erysipelas of the legs and the accompanying worsening of the lower limb lymphedema. At this time, she did not present with edema of the arms (Table 2). Thus, the initial intensive treatment for leg lymphedema was sufficient to treat the arm lymphedema.

This case report was approved by the Research Ethics Committee of FAMERP (# CAAE: 27810214.6.0000.5415).

## 3. Discussion

The present study reports the therapeutic evolution of lymphedema of the four extremities evaluated by lymphoscintigraphy and clinical examination. Physiotherapy was only performed on the lower limbs, but the volume of the upper limbs also reduced. We stress that the arms were not directly treated in the intensive therapy program. A grosgrain stocking was continuously used as a mechanism of contention and mechanical (RA Godoy) and manual lymphatic therapy of the lower limbs (Godoy & Godoy technique) and cervical stimulation (Godoy & Godoy technique) were performed for six to eight hours per day for five days. No study of this type reporting the treatment of an adolescent was found in the literature.

The same therapeutic options as adults are used in the treatment of lymphedema in children, but there are few descriptions in the literature about this age group, mainly in respect to using an intensive form of lymphedema treatment. A recent study describes a new approach to treat lymphedema in children using cervical stimulation thereby adding a new option to the therapeutic arsenal [8].

Lymphedema therapy in children needs to overcome the problems of adolescence, which presents specific therapeutic difficulties. In the current case, the reduction of upper limb edema was maintained for a period of at least four years. This led the adolescent to seek further care when the lower limb lymphedema worsened due to episodes of erysipelas in both legs. The patient continued to perform home treatment to maintain the leg volume as observed during her first return after the intensive treatment program.

The intensive form of treatment used in this teenager has been used by Godoy & Godoy in adults with an average reduction of about 50% of the limb volume within five days [4]. This form of treatment seems to be very important in adolescents, a phase that usually has a series of behavioral problems related to development. The teenager herein described lives about 1500 km away from the treatment center, a fact that made it difficult for her to return frequently. However, she was able to maintain the reduction in the size of the limbs for about four years until she had further episodes of lower limb erysipelas. Another intensive treatment program for five days reduced the size of the limb to near-normal volumes. Frequent monitoring of edema is critical to avoid loss of control; however, intensive treatment for a few days can restore normal or near-normal limb size.

What is remarkable here is the reduction of arm edema without performing any physiotherapy of the upper limbs. The justification for this is the effect of this form of therapy on the system as a whole.

## 4. Conclusion

Isolated treatment of leg lymphedema has systemic repercussions that may lead to a reduction of edema in other untreated areas.

## Figures and Tables

**Table 1:** Volume in liters of the four limbs at 16 years old before and after 5 days of intensive treatment at the Clínica Godoy.

Date	Left leg	Right leg	Left arm	Right arm
March 8, 2010	3.845	3.627	1.256	1.392
March 12, 2010	3.165	2.900	1.251	1.256
Reevaluation April 06, 2010	2.894	2.724	1.293	1.248

**Table 2:** Volume in liters of the four limbs at 20 years old, four years after the intensive treatment at the Clínica Godoy.

Date	Left leg	Right leg	Left arm	Right arm
January 13, 2014	4.025	4.259	1.285	1.281
January 17, 2014	3.226	2.928	1.251	1.255
